# Data on final calcium concentration in native gel reagents determined accurately through inductively coupled plasma measurements

**DOI:** 10.1016/j.dib.2016.01.030

**Published:** 2016-01-29

**Authors:** Jeffrey Viviano, Hao Wu, Anuradha Krishnan, Kandalam Ramanujachary, Venkat Venkataraman

**Affiliations:** a Graduate School of Biomedical Sciences, USA; b School of Osteopathic Medicine, Rowan University, Stratford, NJ 08084, USA; c Department of Chemistry, Rowan University, Glassboro, NJ 08028, USA

**Keywords:** Neuronal calcium sensor proteins, Electrophoresis, Mobility shift, Calcium, Magnesium

## Abstract

In this article we present data on the concentration of calcium as determined by Inductively Coupled Plasma (ICP) measurements. Calcium was estimated in the reagents used for native gel electrophoresis of Neuronal Calcium Sensor (NCS) proteins. NCS proteins exhibit calcium-dependent mobility shift in native gels. The sensitivity of this shift to calcium necessitated a precise determination of calcium concentrations in all reagents used. We determined the calcium concentrations in different components used along with the samples in the native gel experiments. These were: 20 mM Tris pH 7.5, loading dye and running buffer, with distilled water as reference. Calcium determinations were through ICP measurements. It was found that the running buffer contained calcium (244 nM) over the blank.

**Specifications table**

**Table:** 

Subject area	*Biology*
More specific subject area	*Electrophoresis, Calcium measurements*
Type of data	*Table*
How data was acquired	*Inductively Coupled Plasma:* Agilent 7500 series ICP-MS
Data format	*Analyzed*
Experimental factors	*For ICP, standard protocols were used*
Experimental features	*The divalent cations calcium and magnesium were measured in the reagents used for native gel electrophoresis*
Data source location	*Not Applicable*
Data accessibility	*Data is within this article*

**Value of the data**

• Calcium is ubiquitous in most solutions.
• Accurate calcium measurements are critical for a wide variety of applications both on the laboratory bench and by the bedside of the patient.
• Electrophoresis is a commonly used, yet powerful, laboratory technique.
• NCS proteins exhibit a calcium-dependent mobility shift in native gels, which is very sensitive to calcium.
• Accurate determination of calcium concentrations in these experiments is essential for physiologically relevant conclusions to be made.

## 1 Data

The calcium-dependent mobility shift of NCS proteins were determined by loading the proteins in native gels and subjecting them to electrophoresis. Apart from the protein and calcium calibration buffer, the loaded sample contained additional components which were tested in this report. They are 20 mm Tris pH 7.5 (Chelex100-treated), loading dye and running buffer, and distilled water treated with Chelex-100 (Chelex-water). Samples and standards were diluted in 5% nitric acid in ICP grade water rated at 18.2 MΩ cm. Data was compiled from at least three independent replicates for each reagent.

## 2 Experimental design, materials and methods

1. Concentration of calcium in native gel reagents

**Table:** 

**Sample**	**Total volume in sample (%)**	**[Ca**^**2+**^**] In μM**
Chelex-water	0	BLANK
20 mM Tris pH 7.5 (Chelex-treated)	17	–
Running Buffer	17	1.464±0.174
Loading Dye	17	–
Total per Lane	100	0.244±0.029

Mobility Retardation of NCALD (and other NCS proteins) in native gels has been documented and the retardation was directly dependent on the concentration of calcium [1], [2]. Electrophoreses in native gels and analyses were carried out as described [1]. Extraneous calcium in solutions was removed through treatment with Chelex-100 resin (BioRad Laboratories, CA, USA) using standard procedures. In order to determine if divalent cations such as calcium were still carried over in solutions, ICP analyses were carried out. Components other than the protein and calcium calibration buffer in the sample loaded for electrophoresis are: 20 mM Tris pH 7.5 (Chelex-treated), loading dye (63 mM Tris HCl, 0.1%, betamercaptoethanol, 0.0005% bromophenol blue, 10% Glycerol) and running buffer (25 mM Tris, 192 mM glycine). The concentration of calcium in these components was determined by ICP. Chelex-treated water served as a blank. Briefly, samples were diluted into 10 mL of 5% nitric acid in ICP grade water rated at 18.2 MΩ cm. The quantity of calcium present was estimated as parts per billion (ppb) and converted into molarity. The data is presented as a table. It is noted that 20 mM Tris pH 7.5 (Chelex-treated) and the loading dye were comparable to Chelex-water, which served as the reference. Only the running buffer contained measurable calcium above the reference.

Binding of magnesium to two NCS proteins, GCAP1 and GCAP2, with functional consequences has been demonstrated [3], [4]. Therefore, the concentration of magnesium in these components was also determined by ICP. Based on the determination, the loaded sample in the native gel experiments contained 0.4169±0.0024 μM magnesium. However, addition of magnesium (even up to 400 μM) has no effect on any tested NCS protein [1], [5].
