# Supplementary material for: Data on final calcium concentration in native gel reagents determined accurately through inductively coupled plasma measurements
Source: Data Brief. 2016 Jan 29;6:820–2. doi: 10.1016/j.dib.2016.01.030 (PMC4749937; doi:10.1016/j.dib.2016.01.030)
Supplement: Supplementary file 1 — Supplementary material [file mmc1.docx]

We wish to confirm that there are no known conflicts of interest associated with this publication and there has been no significant financial support for this work that could have influenced its outcome.

Conflict of Interest: None.
